# NG2 antigen is involved in leukemia invasiveness and central nervous system infiltration in MLL-rearranged infant B-ALL

**DOI:** 10.1038/leu.2017.294

**Published:** 2017-10-17

**Authors:** C Prieto, B López-Millán, H Roca-Ho, R W Stam, D Romero-Moya, F J Rodríguez-Baena, A Sanjuan-Pla, V Ayllón, M Ramírez, M Bardini, P De Lorenzo, M G Valsecchi, M Stanulla, M Iglesias, P Ballerini, Á M Carcaboso, J Mora, F Locatelli, A Bertaina, L Padilla, J Carlos Rodríguez-Manzaneque, C Bueno, P Menéndez

**Affiliations:** 1 Josep Carreras Leukemia Research Institute, Department of Biomedicine, School of Medicine, University of Barcelona, Barcelona, Spain; 2 Erasmus University Medical Center, Rotterdam, The Netherlands; 3 Princess Maxima Center for Paediatric Oncology, Utrecht, The Netherlands; 4 GENYO, Centre for Genomics and Oncological Research: Pfizer/University of Granada/Andalusian Regional Government, Granada, Spain; 5 Oncohematología, Hospital Universitario Niño Jesús, Madrid, Spain; 6 Centro Ricerca Tettamanti, University of Milano-Bicocca, Ospedale San Gerardo Monza, Italy; 7 Interfant Trial Data Center, University of Milano-Bicocca, Monza, Italy; 8 Department of Pediatric Hemato-Oncology, Hannover Medical School, Hannover, Germany; 9 Pathology Service, Hospital del Mar, Barcelona, Spain; 10 Pediatric Hematology, A. Trousseau Hospital, Paris, France; 11 Developmental Tumor Biology Laboratory, Hospital Sant Joan de Deu, Barcelona, Spain; 12 Department of Pediatric Hematology and Oncology, Ospedale Bambino Gesù, Rome, University of Pavia, Pavia, Italy; 13 Biomed Division, LEITAT Technological Centre, Barcelona, Spain; 14 Centro de Investigacion Biomedica en Red-Oncología (CIBERONC), Barcelona, Spain; 15 Instituciò Catalana de Recerca i Estudis Avançats (ICREA), Barcelona, Spain

## Abstract

Mixed-lineage leukemia (MLL)-rearranged (MLLr) infant B-cell acute lymphoblastic leukemia (iMLLr-B-ALL) has a dismal prognosis and is associated with a pro-B/mixed phenotype, therapy refractoriness and frequent central nervous system (CNS) disease/relapse. Neuron-glial antigen 2 (NG2) is specifically expressed in MLLr leukemias and is used in leukemia immunophenotyping because of its predictive value for MLLr acute leukemias. NG2 is involved in melanoma metastasis and brain development; however, its role in MLL-mediated leukemogenesis remains elusive. Here we evaluated whether NG2 distinguishes leukemia-initiating/propagating cells (L-ICs) and/or CNS-infiltrating cells (CNS-ICs) in iMLLr-B-ALL. Clinical data from the Interfant cohort of iMLLr-B-ALL demonstrated that high NG2 expression associates with lower event-free survival, higher number of circulating blasts and more frequent CNS disease/relapse. Serial xenotransplantation of primary MLL-AF4^+^ leukemias indicated that NG2 is a malleable marker that does not enrich for L-IC or CNS-IC in iMLLr-B-All. However, NG2 expression was highly upregulated in blasts infiltrating extramedullar hematopoietic sites and CNS, and specific blockage of NG2 resulted in almost complete loss of engraftment. Indeed, gene expression profiling of primary blasts and primografts revealed a migratory signature of NG2^+^ blasts. This study provides new insights on the biology of NG2 in iMLLr-B-ALL and suggests NG2 as a potential therapeutic target to reduce the risk of CNS disease/relapse and to provide safer CNS-directed therapies for iMLLr-B-ALL.

## Introduction

B-cell acute lymphoblastic leukemia (B-ALL) is the most frequent childhood cancer.^1^ Although better understanding of the disease biology has increased the cure rate of pediatric B-ALL to >80%,^2, 3^ age <1 year (infants) and mixed-lineage leukemia (*MLL*, also known as *KMT2A*) gene rearrangements (MLLr) remain two major adverse prognostic factors in pediatric B-ALL.^4^ Of special interest is the infant B-ALL carrying MLLr (iMLLr-B-ALL), particularly the t(4;11)/MLL-AF4 (MA4, AF4 is also known as *AFF1*), which possesses unique clinico-biological features and represents a subtype of B-ALL with dismal prognosis.^4, 5, 6^ iMLLr-B-ALL has a distinctive pro-B/mixed phenotype (CD10^−^ with expression of myeloid markers) and frequently shows therapy refractoriness and central nervous system (CNS) infiltration.^7^

Treatment strategies aimed at targeting leukemia-initiating/propagating cells (L-IC) and CNS-infiltrating leukemia cells (CNS-IC) are necessary to overcome therapy resistance, relapse and CNS disease.^8, 9^ However, information on the nature of L-IC and CNS-IC in iMLLr-B-ALL is scarce.^4, 10^ The hierarchical stem cell model for B-ALL has been challenged by the demonstration that phenotypically diverse blasts both from MLL-germline and MLLr B-ALL can reproduce the disease in immunodeficient mice.^8, 9, 11, 12^ Moreover, it has recently been reported that the ability to cross the blood–cerebrospinal fluid barrier is a generic property of all B-ALL blasts.^13^ Whether CNS disease/relapse in t(4;11)/MA4 infant B-ALL is driven by some leukemic cells specifically endowed with the ability to enter and seed the CNS (CNS-ICs) or whether it is a stochastic process in which all blasts have the same ability to enter the CNS remains unknown.

Neuron-glial antigen 2 (NG2, also known as *CSPG4*) is a transmembrane proteoglycan barely expressed in normal hematopoietic cells.^14, 15^ By contrast, ~90% of 11q23/MLLr leukemias specifically express NG2, which has been incorporated into diagnostic workflows for leukemia immunophenotyping because of its predictive value for MLLr.^16, 17, 18, 19, 20^ Interestingly, NG2 was originally found highly expressed in melanoma^21, 22^ and associated with tumor cell migration and metastasis.^23, 24, 25^ In addition, NG2 has important role in the developing brain and is expressed by oligodendrocyte progenitors.^26, 27^ Nonetheless, its physiological role in normal hematopoiesis and MLL-mediated leukemogenesis remains unknown.^14, 15^

We have previously suggested that NG2 expression may be dependent on the cell of origin where a specific MLL fusion initially occurs.^14^ This, together with the reported role of NG2 in the developing brain and in tumor cell migration/metastasis, prompted us to hypothesize that NG2 expression in iMLLr-B-ALL may contribute to the CNS disease/relapse frequently observed in B-ALL. Clinical data from the Interfant cohort of iMLLr-B-ALLs showed that high NG2 expression associates with lower event-free survival (EFS), higher numbers of circulating blasts and more frequent CNS disease/relapse. Serial xenotransplantation of primary leukemias indicated that NG2 is a malleable marker that does not enrich for L-IC or CNS-IC; however, its expression was upregulated in MA4^+^ blasts infiltrating extramedullar hematopoietic sites and CNS, which was confirmed by *in vivo* loss-of-function assays and by a migratory transcriptomic signature of NG2^+^ MLLr blasts. Our findings provide new insights on the biology of NG2 in iMLLr B-ALL and encourages further studies to address whether targeting NG2 offers a therapeutic window for CNS disease/relapse.

## Materials and methods

### Patient samples and clinical data

**Figure 1:**
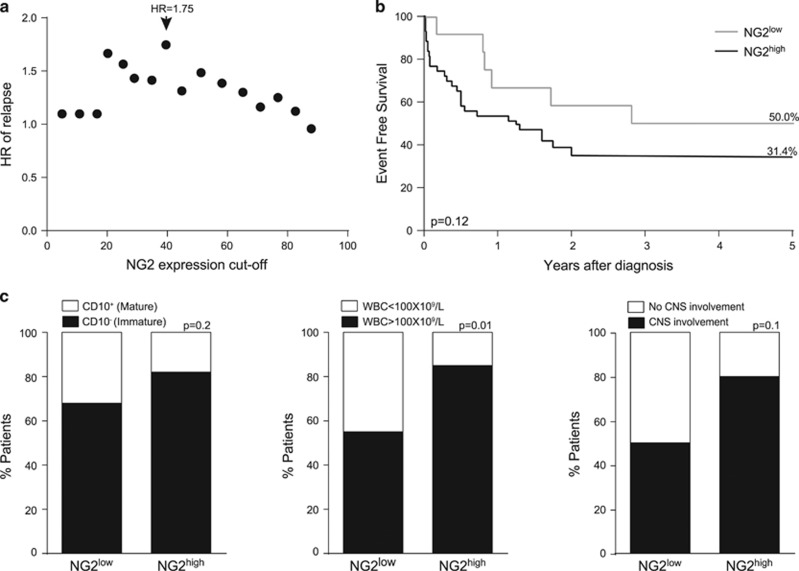
Clinical impact of NG2 expression in MLLr infant B-ALL. (**a**) The HR of relapse for different cutoffs of NG2 expression was investigated to define NG2^high^ versus NG2^low^ patients (*n*=55). The HR of 1.75 corresponding to the 40% cutoff was used. (**b**) Five-year EFS in NG2^high^ and NG2^low^ patients (*n*=55). (**c**) Frequency of patients with immature CD10^neg^ immunophenotype in NG2^high^ and NG2^low^ subgroups (left panel), WBC count at diagnostic (middle panel) and frequency of CNS disease (right panel) in NG2^high^ and NG2^low^ patients (*n*=55).

**Figure 2:**
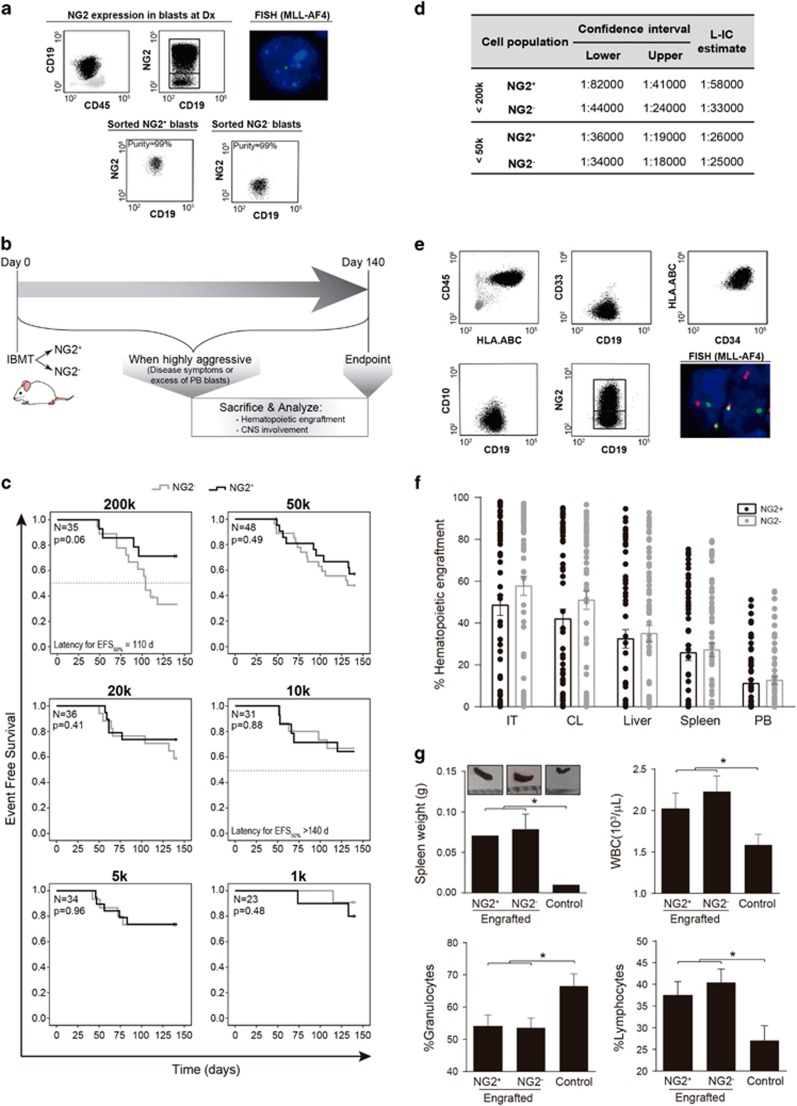
Leukemia development and phenotype in primografts of NG2^+^ and NG2^−^ blast populations. (**a**) Representative NG2 immunophenotype of diagnostic MA4^+^ BM samples and high-purity FACS-sorting of NG2^+^ and NG2^−^ populations. More than 95% of NG2^+^ and NG2^−^ blasts carry the t(4;11)/MA4 by fluorescence *in situ* hybridization (FISH). (**b**) Outline of the *in vivo* experimental design. NG2^+^ or NG2^−^ blasts were IBM-transplanted into NSG mice at day 0. The health of the mice and leukemia development were monitored over 20 weeks. Mice were killed when disease was evident, when leukemic cells were 10% in PB or at day 140 (in the absence of symptoms or PB engraftment). (**c**) Kaplan–Meier survival curves for EFS according to decreasing cell doses (200k to 1k) for NG2^+^ and NG2^−^ transplanted mice (*n*=245). (**d**) Estimated frequency (and 95% confidence interval) of L-IC in NG2^+^ and NG2^−^ primografts calculated for cell doses <200k or <50k. (**e**) Representative flow cytometric analysis of leukemic mice. The human graft, identified as CD45^+^HLA-ABC^+^, reproduces the pro-B phenotype (CD34^+^CD19^+^CD10^−^) seen in patients. Engrafted leukemias always re-establish NG2 variable expression and carry the t(4;11)/MA4 as detected by dual-fusion or break-apart FISH. (**f**) Level of leukemia engraftment in hematopoietic tissues from mice transplanted with NG2^+^ and NG2^−^ blasts. Each dot represents a transplanted mouse and bars represent mean level of engraftment. (**g**) Both NG2^+^ and NG2^−^ transplanted mice consistently displayed splenomegaly, high WBC counts and a skewed granulocytic-to-lymphoid cell representation in PB. Control group includes non-engrafted mice. *P<0.05.

Clinical data was available for 67 diagnostic iMLLr-B-ALLs. Fifty-five of these infants were enrolled in the Interfant treatment protocol.^28^ Clinico-biological correlations in Figure 1 are based on the Interfant cohort except CNS disease data, which was obtained from 12 iMLLr-B-ALL used for xenotransplantation studies. Table 1 shows the clinico-biological data of the patients contributing samples for the experiments. The Institutional Review Board of the Hospital Clinic of Barcelona approved the study, and all patients’ parents gave written informed consent. Mononuclear cells from patients with >85% of blasts were isolated from diagnostic bone marrow (BM) or peripheral blood (PB) by density gradient centrifugation using Ficoll-Hypaque. Blasts were FACS (fluorescence-activated cell sorter)-immunophenotyped using the monoclonal antibodies CD45-FITC, CD19-APC, CD10-PerCP-Cy5.5, CD34-PE-Cy7 (BD Biosciences, San Jose, CA, USA) and NG2-PE (Beckman, Barcelona, Spain), and NG2^+^ and NG2^−^ blast populations were FACS-sorted (FACS Aria, San Jose, CA, USA) (Figure 2a).

### Mice transplantation and follow-up

Eight-to-14-week-old non-obese diabetic/LtSz-scid IL-2Rγ^−/−^ mice (NSG; *n*=305 with male and female equally distributed) housed under pathogen-free conditions were used. All experimental procedures were approved by the Animal Care Committee of The Barcelona Biomedical Research Park (DAAM7393). Limiting dilution doses (200k, 50k, 20k, 10k, 5k and 1k) of sorted NG2^+^ and NG2^−^ leukemic blasts were intra-BM (IBM)-transplanted into sublethally irradiated mice as previously described.^29, 30, 31^ PB was collected weekly to analyze leukemia engraftment by flow cytometry. Mice were killed when (i) disease symptoms were evident, (ii) leukemia engraftment reached 10% in PB, or (iii) 140 days after transplantation. For secondary transplantation, BM-derived mononuclear cells were collected from primografts and IBM-transplanted (200k and 10k doses) into irradiated secondary recipients (*n*=47) as above. For intravenous (IV) transplantation (*n*=15), 200k and 50k NG2^+^ or NG2^−^ sorted blasts were transplanted via the lateral tail vein as described.^32^ IV-transplanted mice were killed and analyzed when human chimerism was detectable in PB.

### NG2 blocking experiments

In order to confirm the role of NG2 in extramedullary infiltration, NG2^+^ primary blasts (200 000 per mouse) were incubated overnight with (i) Chondroitinase (Chase) ABC, proteoglycan-degrading enzyme^33, 34^ (0.1 U/ml, Sigma, Madrid, Spain) or two different anti-NG2 monoclonal antibodies (MoAb), (ii) the clone 7.1 (0.7 mg/ml, 7.1 MoAb-producing hybridoma kindly provided by Professor Irwin Bernstein, Fred Hutchinson Cancer Centre, Seattle, WA, USA) or (iii) the clone 9.2.21 (100 μg/ml), Abcam, Cambridge, UK). NG2-blocked leukemic blasts were IV-transplanted into irradiated NSG mice (*n*=29 mice) and leukemia engraftment analyzed 6 weeks later.

### Analysis of leukemia engraftment and PB hematological counts

BM from injected tibia (IT), contralateral tibia (CL), liver, spleen and PB were collected and analyzed at killing. Cells were stained with HLA-ABC-FITC and CD45-APC-Cy7 antibodies to identify human leukemia by flow cytometry. Leukemia was immunophenotyped using CD19-V450, CD10-PerCP-Cy5.5, CD34-PE-Cy7, CD33-APC and NG2-PE antibodies. Absolute white blood cell counts (WBC) and differential counts were determined in PB.^35^ Hepatosplenomegaly was analyzed as described.^36^ The t(4;11)/MA4 rearrangement was confirmed by dual-fusion fluorescence *in situ* hybridization in both diagnostic NG2^+^ and NG2^−^ cell populations and in engrafted leukemic cells as described.^37, 38^ Additionally, the cell cycle distribution of NG2+ and NG2^−^ leukemic blast was carried out by FACS (*n*=2) using propidium iodide as described.^35, 39^

### Analysis of CNS infiltration

Mice skulls were retrieved at killing, fixed, decalcified, embedded in paraffin and cut-stained with hematoxylin and eosin as previously described.^36^ Ten skull sections/mouse (*n*=60 mice) were analyzed and classified by the presence/absence of infiltrating blasts. Human chimerism in the skull was assessed by immunohistochemistry using the Benchmark automated staining instrument and the human antibodies CD19 and CD45 (Roche, Madrid, Spain).

NG2 expression in CNS-ICs was analyzed by immunofluorescence in skull sections, using spleen as a positive control. Primary antibodies used were rabbit anti-NG2 and rat anti-endomucin (Millipore, Madrid, Spain). Secondary antibodies used were donkey anti-rabbit and anti-rat (Life Technologies, Madrid, Spain). Human NG2 expression was confirmed by quantitative reverse transcriptase-PCR (qRT-PCR) in BM and extramedullar tissues. RNA from the BM, liver and spinal cord of engrafted mice was cDNA-converted and used for RT-PCR as previously described^36^ using the following primers: NG2; Fwd-5′-CCTCTGGAAGAACAAAGGTCTC-3′, Rev-5′-GAACTGTGTGACCTGGAAGAG-3′ and GAPDH (glyceraldehyde 3-phosphate dehydrogenase), Fwd-5′-GGGAAGCTTGTCATCAATGGA-3′, Rev-5′-CGCCCCACTTGATTTTGG-3′. PCR conditions were 95 °C (20 s) followed by 40 cycles of 95 °C (1 s) and 60 °C (20 s).

### Microarray gene expression profiling

CD34^+^CD19^+^CD10^−^NG2^+^ and CD34^+^CD19^+^CD10^−^ NG2^−^ blast populations were FACS-purified from the BM of three iMLLr-B-ALL for global gene expression profiling (GEP) as described.^40^ Hierarchical clustering of genes was performed with the one-minus-correlation metric and the unweighted average distance. Gene functions and canonical pathways was analyzed using the Ingenuity Pathway Analysis (IPA) software. Microarray data were deposited in the public Gene Expression Omnibus database, accession number GSE19475.

To confirm gene expression changes, a qPCR array was used to analyze the expression of 84 genes involved in epithelial-to-mesenchymal transition/migration pathways (QIAGEN, Madrid, Spain). We specifically compared circulating NG2^+^ and NG2^−^ blasts recovered from primografts transplanted with NG2^+^ versus NG2^−^ cells. PCR Array was performed on a Stratagene-Mx3000P System (San Diego, CA, USA) following the manufacturer’s instructions. Raw data were analyzed using the SABiosciences (Madrid, Spain) web-based tool. Genes showing >1.5-fold-change expression were considered differentially expressed between both groups. IPA software was used to predict top regulated pathways/gene functions.^41^

### Statistical analysis

Data are expressed as mean±s.e. of independent experiments unless otherwise specified. Statistical comparisons were performed using either paired or unpaired Student’s *t*-test, as appropriate. Fisher’s exact test was used to assess the association between clinical characteristics and NG2 expression (high versus low levels). Statistical significance was defined as *P*-value<0.05. EFS was calculated as the time from diagnosis to first failure (induction failure, relapse, death or second neoplasm). EFS curves of patients and xenografts were estimated according to Kaplan–Meier and compared with the log-rank test. The Cox model was used to estimate the impact of NG2 expression on the cause-specific hazard-of-relapse. Analyses were performed with the SPSS Software (Hong Kong, China). L-IC frequency was calculated using the ELDA software (http://bioinf.wehi.edu.au/software/elda/) based on limiting dilution transplantation assays.^42^

## Results

### High levels of NG2 are associated with a more immature/aggressive phenotype in iMLLr-B-ALL

The clinical impact of NG2 expression was analyzed in an Interfant cohort (*n*=55) with the exception of CNS disease data that was analyzed in the cohort (*n*=12) of iMLLr-B-ALL (mainly MA4) available for xenotransplantation (Table 1). Percentages of NG2 expression in the blast population (5–90%) were explored as potential thresholds for the definition of high (NG2^high^) versus low (NG2^low^) levels of NG2 expression and the outcome in these subgroups evaluated in terms of risk-of-relapse. The cutoff of 40% NG2 expression was associated with the highest hazard ratio (HR) of relapse, that is, 1.75, suggesting that patients with NG2 expression ⩾40% (NG2^high^) had a 75% increase in the hazard-of-relapse as compared with patients with NG2 expression <40% (NG2^low^) and was thus chosen as the cutoff for NG2 subgroup definition in further analyses (Figure 1a). Accordingly, NG2^high^ infants showed a biologically/clinically relevant but no statistically significant trend toward a more aggressive phenotype (31%±9 versus 50%±10 5-year EFS±s.e., *P*=0.1, Figure 1b) than NG2^low^ patients that was associated with a more immature (CD10^−^ or byphenotypic) phenotype (82% versus 68%, *P*=0.2, Figure 1c, left panel), higher WBC (85% versus 55% of patients with >100 × 10^9^ WBC/l, *P*=0.01, middle panel) and more common CNS disease (80% versus 50% of patients with CNS disease, *P*=0.1, right panel).

### NG2 is a malleable marker that does not enrich for L-IC in iMLLr-B-ALL

Increasing evidence based on maturation-dependent antigens, including CD34, CD10 and CD20, suggests that there is no stem cell hierarchy in pediatric B-ALL.^8, 9, 11^ NG2 is specifically expressed in MLLr leukemia but its function remains enigmatic.^14, 15^ Here we addressed whether NG2 expression defines a blast population enriched in L-IC activity. The ability of highly purified (FACS purity >98%, Figure 2a) NG2^+^ and NG2^−^ blasts to initiate leukemia was interrogated by IBM transplantation into NSG mice (*n*=243) following a limiting dilution (200k–1k blasts) transplantation approach summarized in Figure 2b. The majority (83%) of iMLLr samples were able to transfer the leukemia onto primografts (Table 1, Supplementary Table S1). As expected, EFS decreased with increasing doses of blasts transplanted (Figure 2c); however, with the exception of a slightly (*P*=0.06) more aggressive behavior of NG2^−^ blasts transplanted at very high dose (200k), no differences were observed in either EFS or frequency of engrafted mice between NG2^+^ and NG2^−^ populations injected at decreasing cell doses (Figure 2c, Supplementary Table S1). The estimated frequency of LIC was similar between NG2^+^ and NG2^−^ populations (Figure 2d). Importantly, both NG2^+^ and NG2^−^ engrafted leukemias remained MLLr and mirrored the original pro-B phenotype (CD45^+^CD19^+^CD10^−^) (Figure 2e), with engrafted mice presenting very similar BM and extramedullary hematopoietic site infiltration (Figure 2f), splenomegaly, high WBC counts and a skewed granulocytic-to-lymphoid cell representation in PB (Figure 2g). Interestingly, NG2 expression was malleable as determined by the ability of both NG2^+^ and NG2^−^ populations to re-establish *in vivo* the original leukemia immunophenotype with a continuum expression of NG2 (Figures 2a and e).

**Figure 3:**
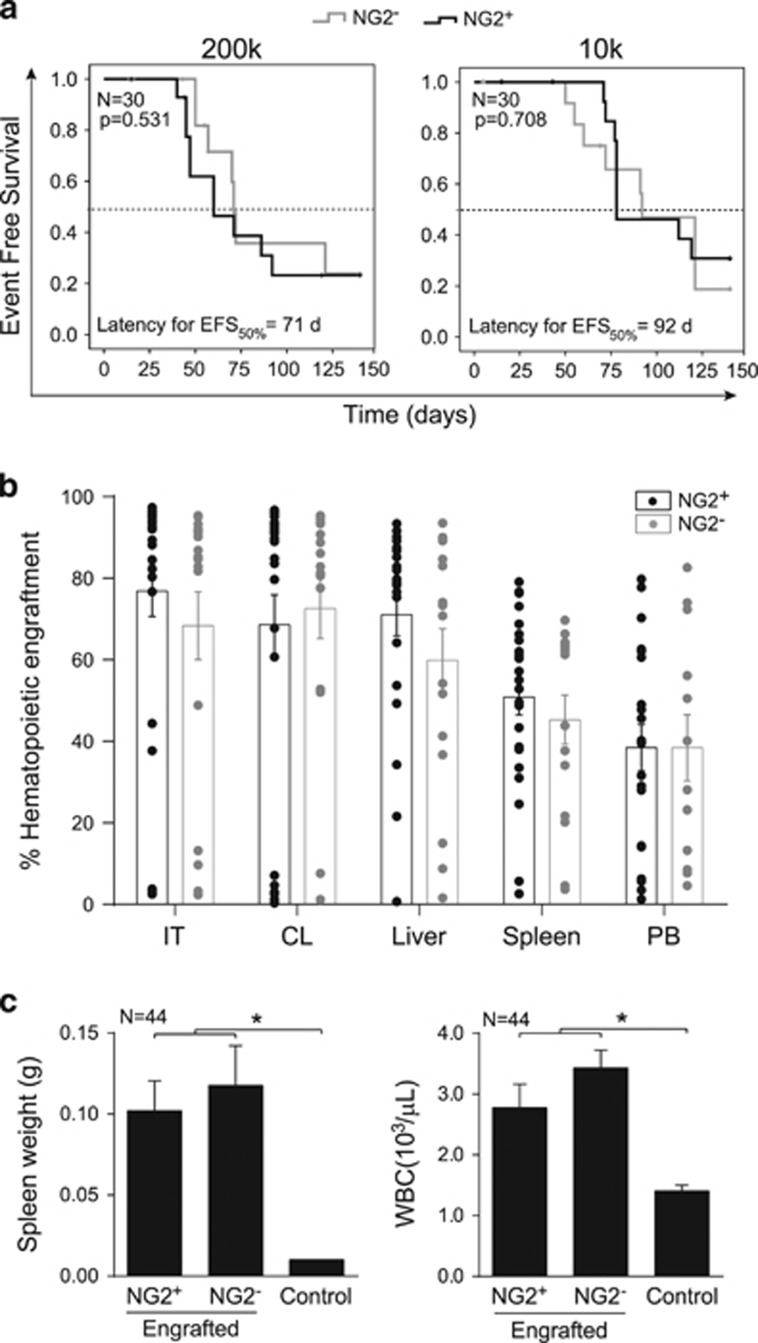
NG2 expression does not enrich for L-IC capacity in secondary recipients. (**a**) Kaplan–Meier survival curves for EFS according to different cell doses transplanted into secondary recipients (*n*=60). Black and gray lines represent secondary recipients transplanted with NG2^+^ and NG2^−^ primary animals, respectively. Dotted line depicts EFS rate of 50%. (**b**) Percentage of long-term leukemic engraftment in the injected (IT) and contralateral (CL) tibia, liver, spleen and PB of secondary mice. (**c**) Secondary recipients of cells from either NG2^+^ or NG2^−^ primary mice consistently displayed splenomegaly and high WBC counts.*P<0.05.

For serial transplantation experiments, 200k or 10k leukemic cells from primografts were transplanted into secondary mice, rendering a lower EFS (higher aggressiveness, Figure 3a, Supplementary Table S2) than that observed in primary recipients; however, no differences in EFS were observed between mice transplanted with NG2^+^ or NG2^−^ primografts (Figure 3a), suggesting an overall enrichment of L-ICs in secondary recipients irrespective of NG2 expression. Importantly, secondary leukemias from NG2^+^ and NG2^−^ primografts retained a pro-B phenotype (Supplementary Figure S1A), similar infiltration levels in BM and extramedullary hematopoietic sites (Figure 3b), splenomegaly, high WBC counts (Figure 3c) and skewed differentiation toward the lymphoid compartment in PB (Supplementary Figure S1B). Of note, the continuum NG2 expression was reproduced in secondary mice (Supplementary Figure S1A), demonstrating the malleability of NG2 expression.

### NG2 is upregulated in extramedullary hematopoietic tissues in iMLLr-B-ALL

**Figure 4:**
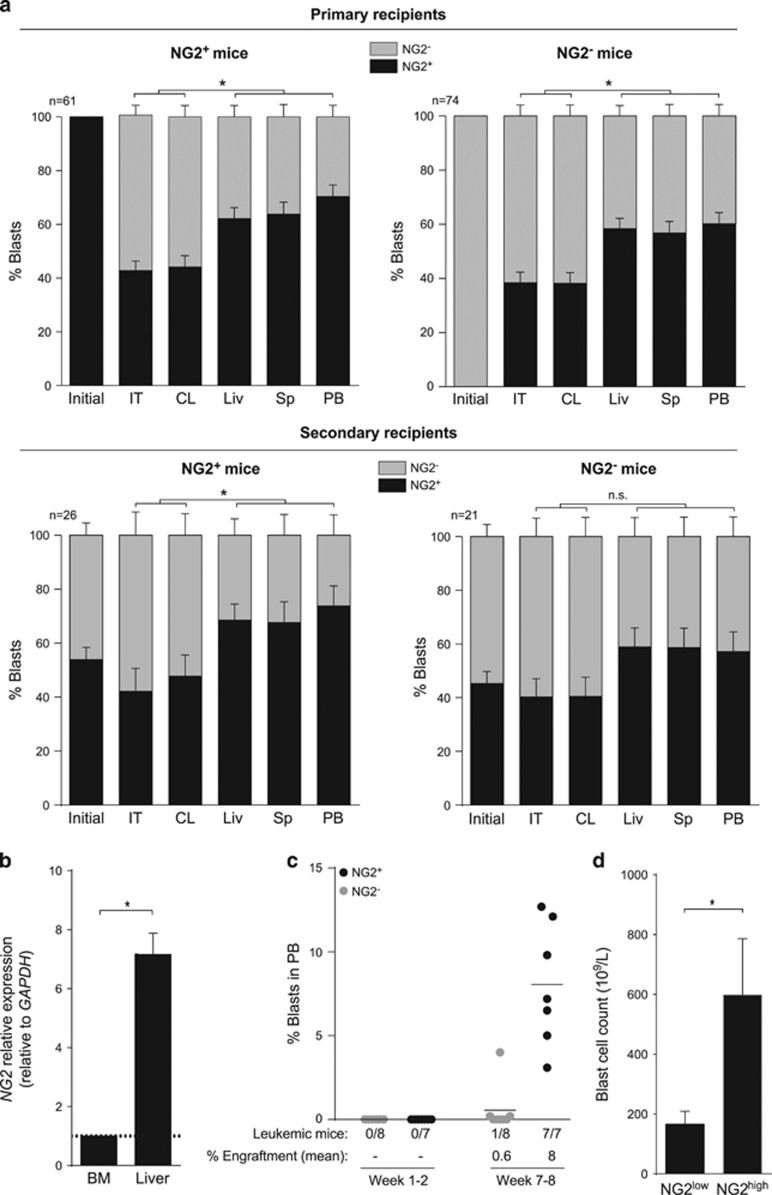
NG2 expression is upregulated in extramedullary hematopoietic tissues. (**a**) Percentage of NG2-expressing blasts (black bar) in primary (top panels) and secondary (bottom panels) engrafted mice at killing (*n*=245). The following tissues were analyzed: IT, intra-tibia; CL, contralateral tibia; Liv, liver; Sp, spleen; PB, peripheral blood. Left panels, NG2^+^ mice. Right panels, NG2^−^ mice. The initial condition represents the percentage of NG2-expressing cells at the moment of transplantation: 100% for primary NG2^+^ mice (black bar) and 0% for primary NG2^−^ mice (gray bar). (**b**) RT-qPCR confirming sevenfold higher expression of NG2 in xenografted liver as compared with BM. (**c**) NG2^+^ and NG2^−^ cells were sorted and IV injected. Mice were weekly monitored by FACS for chimerism and killed after 7–8 weeks. Leukemia engraftment in PB is shown for NG2^+^ (black dots) and NG2^−^ (gray dots) transplanted mice. (**d**) Threefold higher WBC counts in diagnostic NG2^high^ versus NG2^low^ MLL-AF4^+^ infants. Mean of NG2^+^ cells was used as cutoff (Table 1). *P<0.05; NS, not significant.

Despite the IBM transplantation of a pure population of NG2^+^ or NG2^−^ blasts, all leukemic cells engrafting primary and secondary mice showed a re-establishment of the NG2 phenotype, reproducing the continuum observed in the original leukemia (Figures 2e, 3a and 4a). However, NG2 expression followed a tissue-specific pattern upon primary and secondary transplantation (Figure 4a). Whereas ~40% of engrafted blasts expressed NG2 in BM, ~65% of blasts were NG2^+^ in extramedullary hematopoietic tissues (*P*=0.01,Figure 4a). This was confirmed by qRT-PCR, showing sevenfold higher NG2 expression in extramedullary sites than in BM (Figure 4b). Because leukemia reconstitution requires proper homing to and seeding in the BM followed by exit of proliferating blasts to extramedullary organs, NG2^+^ and NG2^−^ blasts were IV-transplanted and engraftment was analyzed 8 weeks later. IV-transplanted NG2^+^ blasts engrafted extramedullary tissues in 100% of mice (8% reconstitution levels) while NG2^−^ blasts did so only in 13% of recipients with ~0.6% reconstitution levels (Figure 4c). Importantly, analysis of leukocytosis in PB of iMLLr-B-ALL patients showed that NG2^high^ iMLLr-B-ALL infants displayed a striking threefold higher number of circulating blasts than NG2^low^ patients (*P*=0.01, Figure 4d). Together, these data suggest that NG2 is upregulated in response to systemic infiltration/migration, which is suggestive of a homeostatic adaptation of leukemic cells.

### NG2 is not a prospective marker for CNS-IC but is upregulated in almost all MLLr blasts entering the CNS

**Figure 5:**
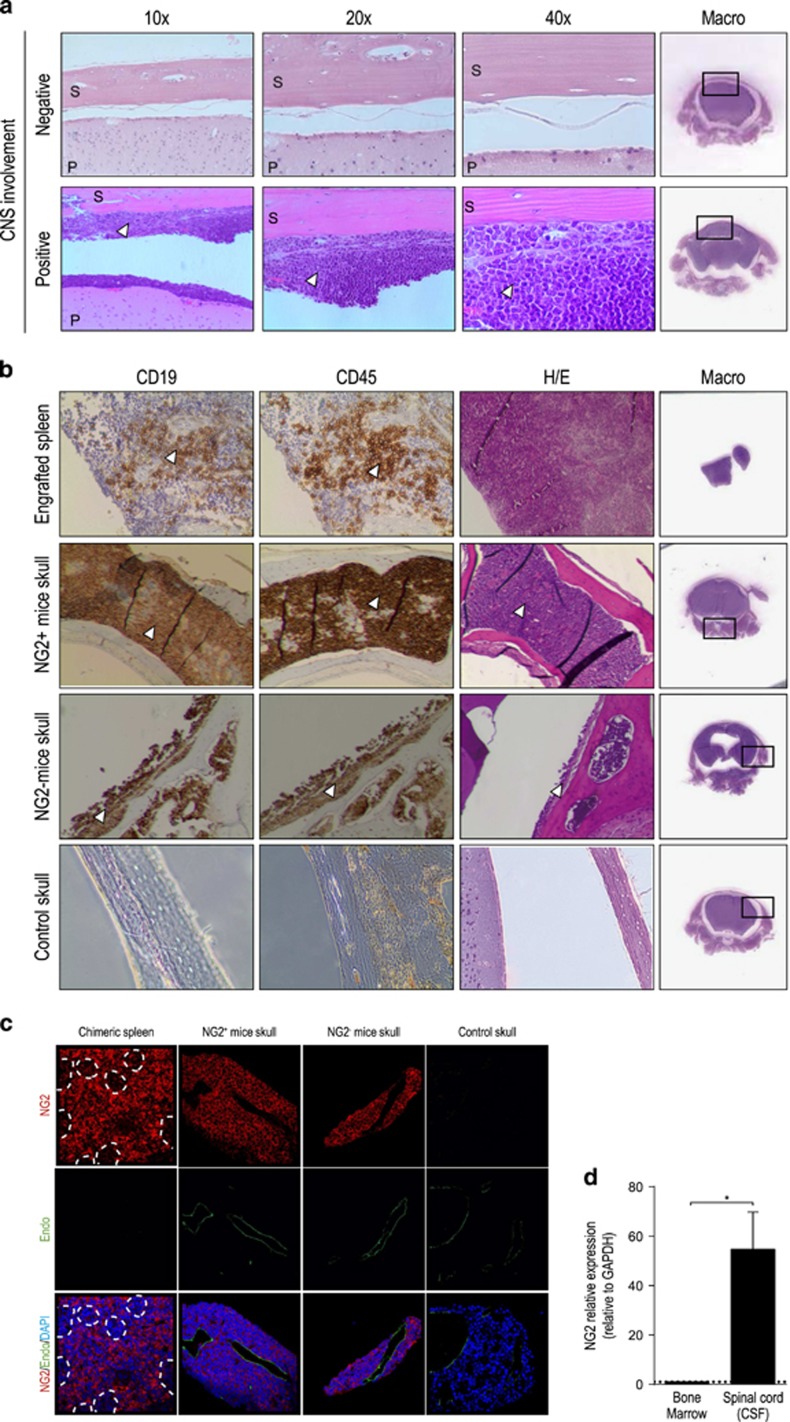
NG2 is not a prospective marker for CNS-IC but it is expressed in almost all MLLr blasts entering the CNS. (**a**) Representative H&E staining of mice brains defining xenografts with negative and positive CNS involvement (*n*=60). S, skull. P, brain parenchyma. Leukemic infiltration is exclusively found in leptomeninges and is marked by a white arrowhead. The right panel (macro) depicts the area magnified on each row. (**b**) H&E staining and immunohistochemistry for CD19 and CD45 performed on paraffin-embedded skulls from mice transplanted with NG2^+^ and NG2^−^ blasts (*n*=60). Chimeric spleens and skulls from non-engrafted mice were used as positive and negative controls, respectively. CD45^+^CD19^+^ human blasts are marked with a white arrowhead. (**c**) Immunofluorescence staining for NG2, endomucin (endothelial vessel marker) and DAPI on chimeric spleen, NG2^+^ and NG2^−^ transplanted mice skull and control skull (non-engrafted mice). Dot lines delimit areas with high density of cells and low density of NG2 marker in the chimeric spleen. Almost a 100% of human blasts in the CNS are consistently NG2^+^, regardless of the NG2 phenotype of the population transplanted. (**d**) RT-qPCR showing 55-fold higher expression of NG2 in the spinal cord than in BM. *P<0.05.

Three pieces of information led us to address whether NG2 is involved in CNS infiltration by MLLr blasts: (i) MLLr blasts upregulate NG2 expression in extramedullary hematopoietic sites, (ii) CNS infiltration is common in iMLLr-B-ALL and up to 75% of relapses occur within the CNS,^43^ and (iii) NG2 antigen has an important role in the developing brain and is associated with melanoma cell migration and metastasis.^21, 22^ As previously reported, leukemia infiltrates were consistently found in meninges/leptomeningeal space but were rarely found within brain parenchyma^13^ (Figure 5a). The presence of infiltrating human B-lymphoid blasts observed by hematoxylin and eosin staining was always confirmed by histopathology for CD45 and CD19 (Figure 5b). In all, 8/11 (73%) primary leukemias tested for CNS-infiltrating potential reproduced the patient phenotype (Supplementary Figure S2A). Interestingly, 2/8 (25%) patients engrafting CNS in mice showed no CNS involvement throughout disease evolution, indicating that CNS-engrafting capacity seems more prevalent than suggested by diagnostic cerebral-spinal fluid cytospins. Although the frequency of mice displaying CNS disease was identical (50%) between NG2^+^- and NG2^−^-transplanted groups (Supplementary Figure S2B, bottom panel), almost all human blasts reaching the CNS were consistently NG2^+^, irrespective of the NG2 phenotype of the population transplanted (Figure 5c). In line with this result, NG2 expression was 55-fold higher in CNS (spinal cord) than in BM (Figure 5d). These data indicate that, while NG2 is not a marker to prospectively identify CNS-infiltrating ability, it is highly upregulated in MLLr blasts seeding the CNS.

**Figure 6:**
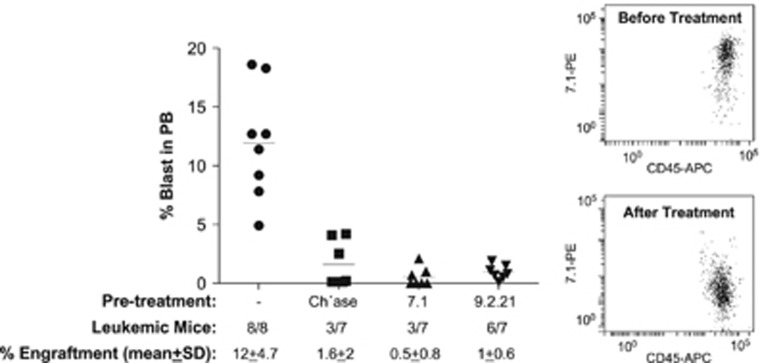
Specific blockage of NG2 antigen with either chase or distinct clones of anti-NG2 MoAb results in dramatic loss of leukemia engraftment (left panel, *n*=29 mice from 2 independent patients). Recovered blasts were mainly negative for NG2 (right panel).

To further confirm NG2 with the migratory phenotype, we next performed functional *in vivo* studies where NG2 antigen was specifically blocked prior to IV xenotransplantation using either the enzyme chase or two different clones of anti-NG2 MoAb (7.1 and 9.2.21). Blockage of NG2 with either compound resulted in a pronounced loss of engraftment by NG2^+^ blasts 8 weeks after transplantation (Figure 6). NG2^+^ blasts engrafted PB of all mice (13%±6% reconstitution levels). whereas NG2-blocked blasts only engrafted 50% of mice with extremely low reconstitution levels (~1%, Figure 6, left panel). Recovered blasts were mainly NG2^−^ (Figure 6, right panel), confirming a direct role of NG2 in invasiveness of MLLr leukemia.

### Global GEP reveals a migratory signature of NG2^+^ MLLr blasts

**Figure 7:**
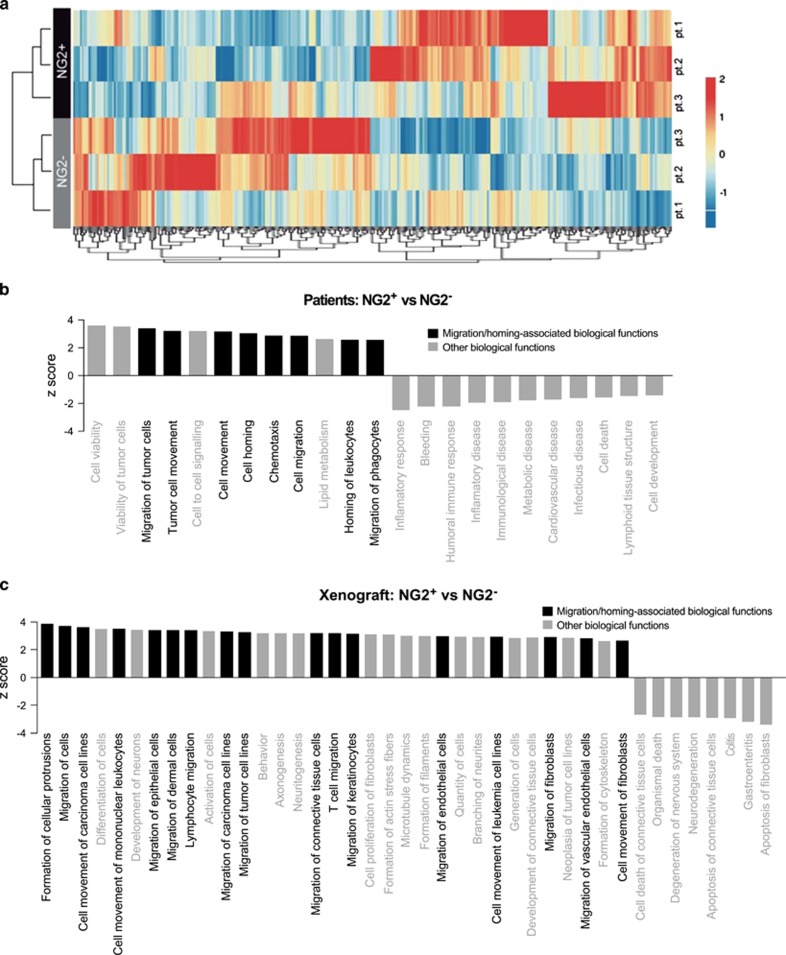
Global GEP reveals a migratory signature of NG2^+^ MLLr blasts. (**a**) Heatmap representation of hierarchical clustering of genes differentially expressed between NG2^+^ and NG2^−^ primary t(4;11)^+^ blasts (*n*=3 independent leukemias). (**b**) Statistically significant biological functions identified using IPA on genes differentially expressed in NG2^+^ versus NG2^−^ blasts. They are ranked by *z*-score. A *z*-score >2 indicates a predicted activation of that biological function. Biological functions associated with ‘leukemic cell viability and migration/movement’ are shown in black. (**c**) Biological functions identified using IPA on genes differentially upregulated in NG2^+^ as compared with NG2^−^ circulating (PB/spleen) blasts in xenografted mice. The RT^2^ profiler PCR array specific for epithelial-to-mesenchymal transition/migration genes was used. Biological functions associated with ‘migration/movement’ are shown in black.

To identify patterns of gene expression that might provide a molecular explanation for the biology of NG2 expression in MLLr, we performed whole-genome GEP on FACS-purified NG2^+^ and NG2^−^ primary cells from t(4;11)/MA4^+^ pro-B ALL infants. A heatmap representation of hierarchical clustering of genes differentially expressed (20% upregulated or downregulated; *P*<0.05) between NG2^+^ and NG2^−^ primary blasts is represented in Figure 7a. A total of only 281 genes (4%) were differentially expressed between NG2^+^ and NG2^−^ primary t(4;11)^+^ blasts. Of these, 142 (50.5%) were upregulated and 139 (49.5%) downregulated in NG2^+^ cells, indicating little transcriptomic differences between both cell subsets. To get insight into the biological functions affected by differentially expressed genes, we used the IPA software^44, 45, 46^ to compare NG2^+^ and NG2^−^ primary t(4;11)^+^ blasts. We found that 8/12 (67%) significant biological processes predicted to be activated in the NG2^+^ blasts were associated with ‘leukemic cell migration/movement’ (Figure 7b), which is compatible with the functional upregulation of NG2 observed in MLLr blasts infiltrating extramedullary tissues and CNS.

To functionally support and validate patient GEP data, we performed qPCR to quantify the expression of a panel of genes associated with epithelial-to-mesenchymal transition/migration in NG2^+^ and NG2^−^ blasts harvested from primografts. Differentially expressed genes were analyzed by IPA, and we found that the majority (33/41, 81%) of the significant biological processes were associated with ‘cell migration/movement’ and were predicted to be activated (*z*-score >2) in NG2^+^ circulating blasts (Figure 7c), further confirming a migratory signature observed in NG2^+^ MLLr blasts infiltrating extramedullary hematopoietic tissues and CNS.

## Discussion

MLLr is associated with poor outcome in acute myeloid leukemia and B-ALL.^4, 47^ A clinico-biologically intriguing MLLr is MA4, which results from the t(4;11)(q21;q23), and is the hallmark genetic abnormality of infant t(4;11)^+^ pro-B/mixed B-ALL. Infant MA4^+^ B-ALL has a dismal prognosis associated with therapy refractoriness and CNS disease/relapse.^6, 48^ Unfortunately, current disease models fail to faithfully recapitulate the disease phenotype, challenging *in vivo* studies aimed at understanding the disease pathogenesis.^4^ Indeed, the nature of the target cell for transformation, the L-IC and the CNS-IC in iMLLr-B-ALL remans unkonwn. Here we analyzed whether NG2 segregates L-ICs and/or CNS-ICs in iMLLr-B-ALL (mainly MA4^+^ infants). NG2 is not expressed in normal hematopoietic cells but it is specifically expressed in 11q23/MLLr leukemias, reason for which it is currently used in immunophenotyping panels for its predictive value in leukemia diagnosis and minimal residual disease studies.^16, 17, 18, 19, 20^

Longstanding evidence justifies the biological interest in NG2 as a potential surface marker involved in leukemia propagation and CNS involvement.^14, 15^ We have previously demonstrated that NG2 expression may be dependent on the cell of origin where a specific leukemic abnormality occurs.^14^ For instance, NG2 might be specifically regulated when leukemic drivers arise either in a specific lineage-committed progenitor or in a more immature hematopoietic stem cells. Furthermore, iMLLr-B-ALL patients commonly show CNS disease either at presentation or at relapse,^43^ NG2 has a role in the developing brain and is associated with melanoma cell migration/metastasis.^21, 22^ Our functional data following a limiting dilution approach in NSG mice serially transplanted with highly purified NG2^+^ or NG2^−^ MA4^+^ blasts confirms the high-risk evolution of MA4^+^ iB-ALL since as many as 83% and 100% of patient samples are able to transfer the leukemia onto primografts and secondary recipients, respectively, despite transplanting a limited number of cells. Interestingly, however, while NG2 does not enrich for L-IC or CNS-IC in iMLLr-B-ALL, its expression is malleable as determined by the ability of both NG2^+^ and NG2^−^ cell fractions to re-establish *in vivo* the original leukemia immunophenotype with a continuum of NG2 expression. This data, together with previous work from several laboratories using other maturation-dependent antigens, including CD34, CD10 and CD20, support the notion that high-risk MLLr-B-ALL does not follow a stem cell hierarchy in pediatric/infant B-ALL.^8, 9, 11^ Recent findings from Vormoor laboratory support our data as they found that NG2 was not expressed in MLLr CD34^+^CD38^−^ hematopoietic stem cells but was upregulated in differentiated CD33^+^ and CD19^+^ MLLr acute myeloid leukemia and ALL blasts.^49, 50^

Our study sheds light on the mechanisms of CNS disease/relapse commonly seen in iMLLr-B-ALL. First, NG2 expression does not allow for prospective identification of the CNS-IC in iMLLr-B-ALL, as there is no selective trafficking of NG2^+^ or NG2^−^ cell fractions into the CNS. Second, our data in iMLLr-B-ALL match recent elegant studies by Williams *et al.*^13^ showing that CNS-infiltrating capacity is a generic property of B-ALL blasts. Third, as demonstrated for MLL germline pediatric B-ALL, iMLLr-B-ALL is almost exclusively found in the meninges/leptomeningeal space and rarely in the brain parenchyma, suggesting that B-ALL blasts cross the cerebrospinal fluid barrier rather than the blood–brain barrier. Fourth, our xenograft model recapitulated well CNS disease in iMLLr-B-ALL as CNS engraftment matched in the majority of primary patient xenograft pairs, even when only 1000 primary cells were transplanted. Importantly, CNS engraftment in mice was seen in 8/11 (73%) diagnostic samples; 6/7 (86%) from infants with overt CNS disease and 2/4 (50%) from patients diagnosed to be CNS negative by diagnostic lumbar puncture. In agreement with Williams *et al.*,^13^ we propose that current cytological examination may misdiagnose CNS-negative patients, as many of these have B-ALL blasts capable to colonize and graft the CNS. These findings support that all the children/infants should benefit from prophylactic intrathecal CNS-targeted therapy.

A major contribution of this work is the finding that, while NG2 does not enrich for L-IC or CNS-IC, it constitutes a malleable marker highly upregulated in blasts infiltrating extramedullar hematopoietic sites and CNS in iMLLr-B-ALL. Loss-of-function experiments for NG2 provided a definitive confirmation of its role in migration and engraftment ability of MLLr B-ALL blasts. Our *in vivo* data were supported by GEP studies, revealing a migratory molecular signature observed in NG2^+^ MLLr blasts infiltrating extramedullary hematopoietic tissues and CNS. Clinical data from infants enrolled in the Interfant treatment protocol revealed that NG2^high^ iMLLr-B-ALL patients presented threefold higher number of circulating blasts, suggesting that NG2 is upregulated in response to systemic infiltration/migration, suggestive of a homeostatic adaptation of leukemic cells. Interestingly, we previously reported that RAS mutations cooperate with MA4 to promote extramedullary engraftment and migration of cord blood CD34^+^ hematopoietic stem and progenitor cells^36^; however, here we found no link/correlation between RAS mutations and CNS infiltration. Further genetic and live imaging studies will be necessary to unveil whether NG2 antigen is regulated before BM exit to facilitate migration or, conversely, is regulated in extramedullary tissues/CNS as an immunological surveillance-evading strategy and adaption to foreign splenic/CNS microenvironment.

Clinical data reveals that NG2^high^ iMLLr-B-ALL patients display a trend toward a more immature/aggressive phenotype associated with lower EFS, higher number of circulating blasts and higher CNS relapse. This not only supports the prophylactic use of intrathecal CNS-targeted therapy in all children but should also encourage further studies to address whether targeting NG2 offers a therapeutic window to reduce the risk of CNS disease and/or provide safer CNS-directed therapies. Because NG2 is specifically upregulated in extramedullary hematopoietic sites and CNS but is not enriched in either L-ICs or CNS-IC, these novel therapies should concentrate on effective eradication of NG2^+^ cells that have managed to evade immunological surveillance and have adapted to new microenvironments in extramedullary sites and CNS, rather than targeting selective trafficking or entry mechanisms.

## Figures and Tables

**Table 1:** Clinico-biological features of patients included in this study

*ID*	*Age at dx (months)*	*Gender*	*Cytogenetics*	*RAS status*	*WBC 10*^*3*^*cell/μl*	*CNS disease*	*% blasts NG2+*	*Disease status*	*Mice graft*	*Mice CNS (1°)*	*Mice CNS (2°)*
01	ND	F	MLL-AF4	NA	185	Neg	38	CR	Neg	Neg	ND
02	7	M	MLL-AF4	WT	300	Pos	56	Exitus	Pos	Neg	Pos
03	10	M	MLL-AF10	WT	21	Neg	45	CR	Pos	Pos	Pos
04	6	F	MLL-EPS15	WT	96	Neg	47	CR	Pos	Pos	ND
05	6	F	MLL-AF4	NA	515	Neg	84	Exitus	Pos	Neg	ND
06	1.4	F	MLL-AF4	WT	1332	Pos	59	Exitus	Pos	Pos	Pos
07	6.5	M	MLL-AF4	WT	204	Pos	48	Exitus	Pos	Neg	Pos
08	3.5	M	MLL-AF4	Mut	349	Pos	68	Exitus	Pos	Pos	Pos
09	2	F	MLL-AF4	Wt	263	Pos	42	CR	Pos	Pos	Pos
10	3	F	MLL-AF4	Mut	499	Pos	54	CR	Pos	Neg	ND
11	2	F	MLL-AF4	Mut	57	NA	37	CR	Neg	Neg	ND
12	6.5	F	MLL-AF4	Mut	337	Pos	33	Exitus	Pos	Pos	ND
Mean	4.9	8 F:4 M	10 MLL-AF4; 2 MLL-X	40% mut	351	64%	51	6 CR 6 Exitus	83%	50%	100%

Abbreviations: CR, complete remission; F, female; M, male; MLL, mixed-lineage leukemia; Mut, mutated; NA, non-available; ND, not done; Neg, negative; Pos, positive; WT, wild type/germline.

## References

[bib1] Pui CH, Evans WE. A 50-year journey to cure childhood acute lymphoblastic leukemia. Semin Hematol 2013; 50: 185–196. 23953334 10.1053/j.seminhematol.2013.06.007 PMC3771494

[bib2] Pui CH, Mullighan CG, Evans WE, Relling MV. Pediatric acute lymphoblastic leukemia: where are we going and how do we get there? Blood 2012; 120: 1165–1174. 22730540 10.1182/blood-2012-05-378943 PMC3418713

[bib3] Yeoh AE, Tan D, Li CK, Hori H, Tse E, Pui CH et al. Management of adult and paediatric acute lymphoblastic leukaemia in Asia: resource-stratified guidelines from the Asian Oncology Summit 2013. Lancet Oncol 2013; 14: e508–e523. 24176570 10.1016/S1470-2045(13)70452-2 PMC4059516

[bib4] Sanjuan-Pla A, Bueno C, Prieto C, Acha P, Stam RW, Marschalek R et al. Revisiting the biology of infant t(4;11)/MLL-AF4+ B-cell acute lymphoblastic leukemia. Blood 2015; 126: 2676–2685. 26463423 10.1182/blood-2015-09-667378 PMC4683329

[bib5] Thomas M, Gessner A, Vornlocher HP, Hadwiger P, Greil J, Heidenreich O. Targeting MLL-AF4 with short interfering RNAs inhibits clonogenicity and engraftment of t(4;11)-positive human leukemic cells. Blood 2005; 106: 3559–3566. 16046533 10.1182/blood-2005-03-1283

[bib6] Bueno C, Montes R, Catalina P, Rodriguez R, Menendez P. Insights into the cellular origin and etiology of the infant pro-B acute lymphoblastic leukemia with MLL-AF4 rearrangement. Leukemia 2011; 25: 400–410. 21135858 10.1038/leu.2010.284

[bib7] Pui CH. Acute lymphoblastic leukemia in children. Curr Opin Oncol 2000; 12: 3–12. 10687723 10.1097/00001622-200001000-00002

[bib8] Rehe K, Wilson K, Bomken S, Williamson D, Irving J, den Boer ML et al. Acute B lymphoblastic leukaemia-propagating cells are present at high frequency in diverse lymphoblast populations. EMBO Mol Med 2013; 5: 38–51. 23229821 10.1002/emmm.201201703 PMC3569652

[bib9] le Viseur C, Hotfilder M, Bomken S, Wilson K, Rottgers S, Schrauder A et al. In childhood acute lymphoblastic leukemia, blasts at different stages of immunophenotypic maturation have stem cell properties. Cancer Cell 2008; 14: 47–58. 18598943 10.1016/j.ccr.2008.05.015 PMC2572185

[bib10] Weiland J, Pal D, Case M, Irving J, Ponthan F, Koschmieder S et al. BCP-ALL blasts are not dependent on CD19 expression for leukaemic maintenance. Leukemia 2016 Sep; 30: 1920–1923. 27055873 10.1038/leu.2016.64 PMC4950966

[bib11] Bardini M, Woll PS, Corral L, Luc S, Wittmann L, Ma Z et al. Clonal variegation and dynamic competition of leukemia-initiating cells in infant acute lymphoblastic leukemia with MLL rearrangement. Leukemia 2015; 29: 38–50. 24798483 10.1038/leu.2014.154

[bib12] Jiang Z, Deng M, Wei X, Ye W, Xiao Y, Lin S et al. Heterogeneity of CD34 and CD38 expression in acute B lymphoblastic leukemia cells is reversible and not hierarchically organized. J Hematol Oncol 2016; 9: 94. 27660152 10.1186/s13045-016-0310-1 PMC5034590

[bib13] Williams MT, Yousafzai YM, Elder A, Rehe K, Bomken S, Frishman-Levy L et al. The ability to cross the blood-cerebrospinal fluid barrier is a generic property of acute lymphoblastic leukemia blasts. Blood 2016; 127: 1998–2006. 26869395 10.1182/blood-2015-08-665034

[bib14] Bueno C, Montes R, Martin L, Prat I, Hernandez MC, Orfao A et al. NG2 antigen is expressed in CD34+ HPCs and plasmacytoid dendritic cell precursors: is NG2 expression in leukemia dependent on the target cell where leukemogenesis is triggered? Leukemia 2008; 22: 1475–1478. 18698324 10.1038/leu.2008.134

[bib15] Menendez P, Bueno C. Expression of NG2 antigen in MLL-rearranged acute leukemias: how complex does it get? Leuk Res 2011; 35: 989–990. 21492935 10.1016/j.leukres.2011.03.015

[bib16] Smith FO, Rauch C, Williams DE, March CJ, Arthur D, Hilden J et al. The human homologue of rat NG2, a chondroitin sulfate proteoglycan, is not expressed on the cell surface of normal hematopoietic cells but is expressed by acute myeloid leukemia blasts from poor-prognosis patients with abnormalities of chromosome band 11q23. Blood 1996; 87: 1123–1133. 8562938

[bib17] Behm FG, Smith FO, Raimondi SC, Pui CH, Bernstein ID. Human homologue of the rat chondroitin sulfate proteoglycan, NG2, detected by monoclonal antibody 7.1, identifies childhood acute lymphoblastic leukemias with t(4;11)(q21;q23) or t(11;19)(q23;p13) and MLL gene rearrangements. Blood 1996; 87: 1134–1139. 8562939

[bib18] Hilden JM, Smith FO, Frestedt JL, McGlennen R, Howells WB, Sorensen PH et al. MLL gene rearrangement, cytogenetic 11q23 abnormalities, and expression of the NG2 molecule in infant acute myeloid leukemia. Blood 1997; 89: 3801–3805. 9160687

[bib19] Wuchter C, Harbott J, Schoch C, Schnittger S, Borkhardt A, Karawajew L et al. Detection of acute leukemia cells with mixed lineage leukemia (MLL) gene rearrangements by flow cytometry using monoclonal antibody 7.1. Leukemia 2000; 14: 1232–1238. 10914547 10.1038/sj.leu.2401840

[bib20] Zangrando A, Intini F, te Kronnie G, Basso G. Validation of NG2 antigen in identifying BP-ALL patients with MLL rearrangements using qualitative and quantitative flow cytometry: a prospective study. Leukemia 2008; 22: 858–861. 17851550 10.1038/sj.leu.2404952

[bib21] Price MA, Colvin Wanshura LE, Yang J, Carlson J, Xiang B, Li G et al. CSPG4, a potential therapeutic target, facilitates malignant progression of melanoma. Pigment Cell Melanoma Res 2011; 24: 1148–1157. 22004131 10.1111/j.1755-148X.2011.00929.x PMC3426219

[bib22] Wang Y, Geldres C, Ferrone S, Dotti G. Chondroitin sulfate proteoglycan 4 as a target for chimeric antigen receptor-based T-cell immunotherapy of solid tumors. Expert Opin Ther Targets 2015; 19: 1339–1350. 26190756 10.1517/14728222.2015.1068759

[bib23] Pucciarelli D, Lengger N, Takacova M, Csaderova L, Bartosova M, Breiteneder H et al. Anti-chondroitin sulfate proteoglycan 4-specific antibodies modify the effects of vemurafenib on melanoma cells differentially in normoxia and hypoxia. Int J Oncol 2015; 47: 81–90. 25997619 10.3892/ijo.2015.3010 PMC4485656

[bib24] Gray ES, Reid AL, Bowyer S, Calapre L, Siew K, Pearce R et al. Circulating melanoma cell subpopulations: their heterogeneity and differential responses to treatment. J Invest Dermatol 2015; 135: 2040–2048. 25830652 10.1038/jid.2015.127 PMC4504811

[bib25] Wang X, Wang Y, Yu L, Sakakura K, Visus C, Schwab JH et al. CSPG4 in cancer: multiple roles. Curr Mol Med 2010; 10: 419–429. 20455858 10.2174/156652410791316977

[bib26] Horky LL, Galimi F, Gage FH, Horner PJ. Fate of endogenous stem/progenitor cells following spinal cord injury. J Comp Neurol 2006; 498: 525–538. 16874803 10.1002/cne.21065 PMC2553041

[bib27] Horner PJ, Thallmair M, Gage FH. Defining the NG2-expressing cell of the adult CNS. J Neurocytol 2002; 31: 469–480. 14501217 10.1023/a:1025739630398

[bib28] Pieters R, Schrappe M, De Lorenzo P, Hann I, De Rossi G, Felice M et al. A treatment protocol for infants younger than 1 year with acute lymphoblastic leukaemia (Interfant-99): an observational study and a multicentre randomised trial. Lancet 2007; 370: 240–250. 17658395 10.1016/S0140-6736(07)61126-X

[bib29] Bueno C, Montes R, de la Cueva T, Gutierrez-Aranda I, Menendez P. Intra-bone marrow transplantation of human CD34(+) cells into NOD/LtSz-scid IL-2rgamma(null) mice permits multilineage engraftment without previous irradiation. Cytotherapy 2010; 12: 45–49. 19929453 10.3109/14653240903377052

[bib30] Montes R, Ayllon V, Prieto C, Bursen A, Prelle C, Romero-Moya D et al. Ligand-independent FLT3 activation does not cooperate with MLL-AF4 to immortalize/transform cord blood CD34+ cells. Leukemia 2014; 28: 666–674. 24240202 10.1038/leu.2013.346

[bib31] Romero-Moya D, Bueno C, Montes R, Navarro-Montero O, Iborra FJ, Lopez LC et al. Cord blood-derived CD34+ hematopoietic cells with low mitochondrial mass are enriched in hematopoietic repopulating stem cell function. Haematologica 2013; 98: 1022–1029v. 23349299 10.3324/haematol.2012.079244 PMC3696604

[bib32] Sanjuan-Pla A, Romero-Moya D, Prieto C, Bueno C, Bigas A, Menendez P. Intra-bone marrow transplantation confers superior multi-lineage engraftment of murine aorta-gonad mesonephros cells over intravenous transplantation. Stem Cells Dev 2016; 25: 259–265. 26603126 10.1089/scd.2015.0309

[bib33] Alluin O, Delivet-Mongrain H, Gauthier MK, Fehlings MG, Rossignol S, Karimi-Abdolrezaee S. Examination of the combined effects of chondroitinase ABC, growth factors and locomotor training following compressive spinal cord injury on neuroanatomical plasticity and kinematics. PLoS ONE 2014; 9: e111072. 25350665 10.1371/journal.pone.0111072 PMC4211738

[bib34] Shojaie S, Ermini L, Ackerley C, Wang J, Chin S, Yeganeh B et al. Acellular lung scaffolds direct differentiation of endoderm to functional airway epithelial cells: requirement of matrix-bound HS proteoglycans. Stem Cell Rep 2015; 4: 419–430. 10.1016/j.stemcr.2015.01.004 PMC4375883 25660407

[bib35] Montes R, Ayllon V, Gutierrez-Aranda I, Prat I, Hernandez-Lamas MC, Ponce L et al. Enforced expression of MLL-AF4 fusion in cord blood CD34+ cells enhances the hematopoietic repopulating cell function and clonogenic potential but is not sufficient to initiate leukemia. Blood 2011; 117: 4746–4758. 21389315 10.1182/blood-2010-12-322230

[bib36] Muñoz-López A, Romero-Moya D, Prieto C, Ramos-Mejía V, Agraz-Doblas A, Varela I et al. Development Refractoriness of MLL-Rearranged Human B Cell Acute Leukemias to Reprogramming into Pluripotency. Stem Cell Reports 2016; 7: 602–618. 27666791 10.1016/j.stemcr.2016.08.013 PMC5063541

[bib37] Bueno C, Catalina P, Melen GJ, Montes R, Sanchez L, Ligero G et al. Etoposide induces MLL rearrangements and other chromosomal abnormalities in human embryonic stem cells. Carcinogenesis 2009; 30: 1628–1637. 19587093 10.1093/carcin/bgp169

[bib38] Munoz-Lopez A, Romero-Moya D, Prieto C, Ramos-Mejia V, Agraz-Doblas A, Varela I et al. Development refractoriness of MLL-rearranged human B cell acute leukemias to reprogramming into pluripotency. Stem Cell Rep 2016; 7: 602–618. 10.1016/j.stemcr.2016.08.013 PMC5063541 27666791

[bib39] Prieto C, Stam RW, Agraz-Doblas A, Ballerini P, Camos M, Castano J et al. Activated KRAS cooperates with MLLAF4 to promote extramedullary engraftment and migration of cord blood CD34+ HSPC but is insufficient to initiate leukemia. Cancer Res 2016; 76: 2478–2489. 26837759 10.1158/0008-5472.CAN-15-2769

[bib40] Stam RW, Schneider P, Hagelstein JA, van der Linden MH, Stumpel DJ, de Menezes RX et al. Gene expression profiling-based dissection of MLL translocated and MLL germline acute lymphoblastic leukemia in infants. Blood 2010; 115: 2835–2844. 20032505 10.1182/blood-2009-07-233049

[bib41] Ayllon V, Bueno C, Ramos-Mejia V, Navarro-Montero O, Prieto C, Real PJ et al. The Notch ligand DLL4 specifically marks human hematoendothelial progenitors and regulates their hematopoietic fate. Leukemia 2015; 29: 1741–1753. 25778099 10.1038/leu.2015.74

[bib42] Hu Y, Smyth GK. ELDA: extreme limiting dilution analysis for comparing depleted and enriched populations in stem cell and other assays. J Immunol Methods 2009; 347: 70–78. 19567251 10.1016/j.jim.2009.06.008

[bib43] Evans AE, Gilbert ES, Zandstra R. The increasing incidence of central nervous system leukemia in children. (Children's Cancer Study Group A). Cancer 1970; 26: 404–409. 5271211 10.1002/1097-0142(197008)26:2<404::aid-cncr2820260222>3.0.co;2-i

[bib44] Rodriguez R, Rubio R, Gutierrez-Aranda I, Melen GJ, Elosua C, Garcia-Castro J et al. FUS-CHOP fusion protein expression coupled to p53 deficiency induces liposarcoma in mouse but not in human adipose-derived mesenchymal stem/stromal cells. Stem Cells 2011; 29: 179–192. 21732477 10.1002/stem.571

[bib45] Bueno C, Montes R, Melen GJ, Ramos-Mejia V, Real PJ, Ayllon V et al. A human ESC model for MLL-AF4 leukemic fusion gene reveals an impaired early hematopoietic-endothelial specification. Cell Res 2012; 22: 986–1002v. 22212479 10.1038/cr.2012.4 PMC3367544

[bib46] Rubio R, Gutierrez-Aranda I, Saez-Castillo AI, Labarga A, Rosu-Myles M, Gonzalez-Garcia S et al. The differentiation stage of p53-Rb-deficient bone marrow mesenchymal stem cells imposes the phenotype of *in vivo* sarcoma development. Oncogene 2013; 32: 4970–4980. 23222711 10.1038/onc.2012.507

[bib47] van der Linden MH, Valsecchi MG, De Lorenzo P, Moricke A, Janka G, Leblanc TM et al. Outcome of congenital acute lymphoblastic leukemia treated on the Interfant-99 protocol. Blood 2009; 114: 3764–3768. 19657114 10.1182/blood-2009-02-204214

[bib48] Chillon MC, Gomez-Casares MT, Lopez-Jorge CE, Rodriguez-Medina C, Molines A, Sarasquete ME et al. Prognostic significance of FLT3 mutational status and expression levels in MLL-AF4+ and MLL-germline acute lymphoblastic leukemia. Leukemia 2012; 26: 2360–2366. 22705992 10.1038/leu.2012.161

[bib49] Neudenberger J, Hotfilder M, Rosemann A, Langebrake C, Reinhardt D, Pieters R et al. Lack of expression of the chondroitin sulphate proteoglycan neuron-glial antigen 2 on candidate stem cell populations in paediatric acute myeloid leukaemia/abn(11q23) and acute lymphoblastic leukaemia/t(4;11). Br J Haematol 2006; 133: 337–344. 16643437 10.1111/j.1365-2141.2006.06013.x

[bib50] Hotfilder M, Rottgers S, Rosemann A, Schrauder A, Schrappe M, Pieters R et al. Leukemic stem cells in childhood high-risk ALL/t(9;22) and t(4;11) are present in primitive lymphoid-restricted CD34+CD19- cells. Cancer Res 2005; 65: 1442–1449. 15735032 10.1158/0008-5472.CAN-04-1356

