# Supplementary material for: NG2 antigen is involved in leukemia invasiveness and central nervous system infiltration in MLL-rearranged infant B-ALL
Source: Leukemia. 2017 Oct 17;32(3):633–44. doi: 10.1038/leu.2017.294 (PMC5843903; doi:10.1038/leu.2017.294)
Supplement: Supplementary Figures [file leu2017294x1.docx]

**Figure S1. NG2 expression does not enrich for L-IC capacity in secondary recipients. A**) Representative immunophenotype of leukemias in secondary mice. The human graft, identified as CD45^+^ and HLA.ABC^+^, reproduces the phenotype seen in the primary leukemia and primary recipients: CD34^+^CD19^+^CD10^-^ immature B-lymphoid cells with variable expression of NG2. **B)** Secondary recipients of cells from either NG2^+^ or NG2^-^ primary mice displayed a skewed granulocytic to lymphoid cell representation in PB (n=44). Control mice are non-engrafted mice.* p<0.05

**Figure S2. NG2 is not a prospective marker for CNS-IC but CNS-engrafting capacity is more prevalent than suggested by clinical diagnostic in CSF cytospins. A)** Number (and percentage) of patients showing CNS infiltration at diagnosis (Y-axis) vs number (and percentage) of patient samples developing CNS disease in mice (X-axis). **B)** Percentage of mice displaying CNS involvement according to NG2 phenotype of transplanted blasts. n.s. not significant
