# Supplementary figures and images for: NG2 antigen is involved in leukemia invasiveness and central nervous system infiltration in MLL-rearranged infant B-ALL
Source: Leukemia. 2017 Oct 17;32(3):633–44. doi: 10.1038/leu.2017.294 (PMC5843903; doi:10.1038/leu.2017.294)

**A**

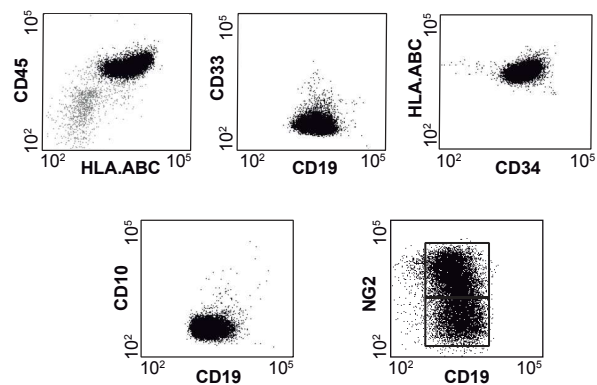

**B**

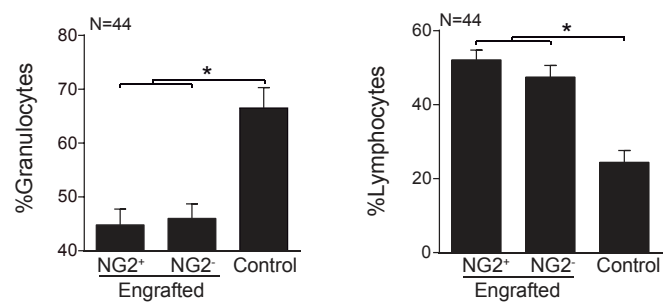

Supplement: Supplementary Figure 1 [file leu2017294x2.pdf]

**A**

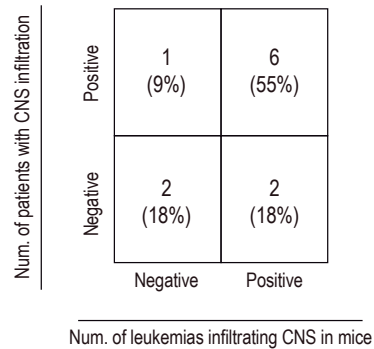

**B**

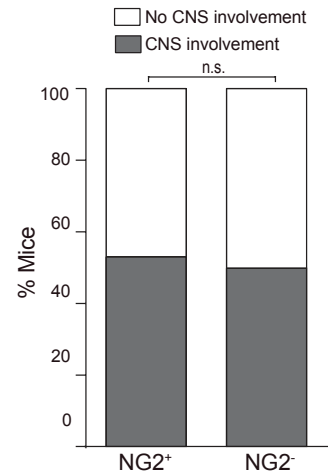

Supplement: Supplementary Figure 2 [file leu2017294x3.pdf]
