# Supplementary material for: NG2 antigen is involved in leukemia invasiveness and central nervous system infiltration in MLL-rearranged infant B-ALL
Source: Leukemia. 2017 Oct 17;32(3):633–44. doi: 10.1038/leu.2017.294 (PMC5843903; doi:10.1038/leu.2017.294)
Supplement: Supplementary Table 2 [file leu2017294x5.docx]

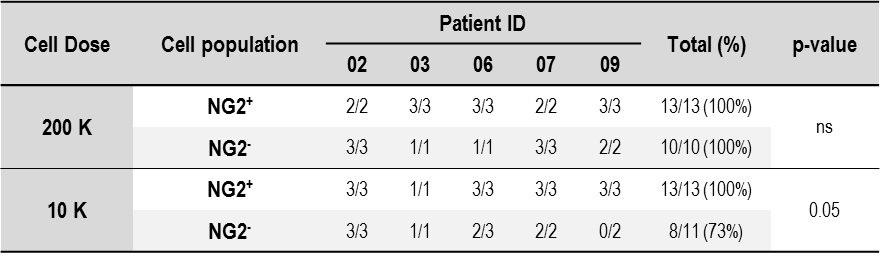


**Table S2:** Number of secondary engrafted mice according to cell dose and cell population transplanted.
